# Pre-Existing Anti-Vector Immunity to Adenovirus-Inspired VLP Vaccines Shows an Adjuvant-Dependent Antagonism

**DOI:** 10.3390/vaccines13030238

**Published:** 2025-02-25

**Authors:** Salomé Gallet, Dalil Hannani, Sebastian Dergan-Dylon, Emilie Vassal-Stermann, Isabelle Bally, Christopher Chevillard, Daphna Fenel, Olivier Epaulard, Pascal Poignard, Pascal Fender

**Affiliations:** 1 CNRS, CEA, IBS, Université Grenoble Alpes, F-38000 Grenoble, France; sgallet@chu-grenoble.fr (S.G.);; 2 Institut de Biologie Structurale, 71 rue des Martyrs, F-38042 Grenoble, France; 3 Clinical Infectious Disease Unit, Grenoble-Alpes University Hospital, F-38000 Grenoble, France; 4 Groupe de Recherche en Infectiologie Clinique, CIC 1406—Inserm—Université Grenoble Alpes, F-38000 Grenoble, France; 5 Université Grenoble Alpes, CNRS, UMR 5525, VetAgro Sup, Grenoble INP, TIMC, F-38400 Grenoble, France

**Keywords:** VLP, adenovirus, adjuvant, pre-existing immunity, SARS-CoV-2

## Abstract

**Background/Objectives:** The use of virus-like particles (VLPs) in vaccinology has expanded significantly in recent years. VLPs have the advantage of being non-infectious while effectively stimulating B cell responses through the repetitive presentation of epitope motifs on their surface. Since VLPs are often derived from human-infecting viruses, preexisting immunity may influence the immune response they elicit, warranting further investigation. **Methods:** We have developed a 60-mer VLP derived from human adenovirus type 3, a common pathogen. We investigated the impact of pre-existing adenovirus immunity on the immunization outcome against the linear S14P5 epitope of SARS-CoV-2, which was engineered into the particle (Ad-VLP-S14P5). To this end, antibody responses to S14P5 were evaluated following immunization with Ad-VLP-S14P5 in either naive or vector-primed mice. **Results:** Mice with pre-existing anti-vector immunity exhibited significantly greater anti-S14P5 antibody responses compared to vector-naive animals, demonstrating a beneficial impact of prior anti-adenovirus responses. However, the addition of an oil-in-water adjuvant for the immunizations abolished this positive impact, even leading to a deleterious effect of the pre-existing anti-vector immunity. **Conclusions:** The data suggest that the immune status against immunizing VLPs must be taken into consideration when designing immunization protocols. Importantly, the effects of prior immunity may vary depending on the nature of the protocol, including factors such as adjuvant use.

## 1. Introduction

Infectious diseases, pandemics, and epidemics can have a devastating impact on the health of populations and the organization of healthcare systems [1,2]. Vaccination is a major tool for both individual and collective protection. However, adaptability is essential for the rapid and effective implementation of this strategy [3,4].

Given their ease of use, absence of genetic material, and thermostability, VLP-based vaccines represent an attractive approach to combat current pandemics and potential future outbreaks [5,6]. Notably, VLPs have been demonstrated to effectively stimulate B cell responses due to the repetitive presentation of epitope motifs on their surface. Depending on the nature of the original biological entity from which they are derived, VLPs can display a variable number of epitopes, ranging from 7 to 180 in different arrangements, with particle sizes varying from 4 to 120 nm [7].

We previously developed a non-infectious 60-mer adenovirus-derived VLP (Ad-VLP), allowing high-density display of linear protein epitopes, such as the E2EP3 epitope of the chikungunya virus [8]. The Ad-VLP is composed of 60 identical units and exhibits a quasi-spherical shape with a diameter of 30 nm, a size allowing efficient drainage to lymph nodes.

Since Ad-VLP is derived from human adenovirus, the potential impact of pre-existing immunity on the response to this vaccine platform must be considered, as for any VLPs derived from viruses infecting humans [9,10]. Adenoviruses, one of the main causes of winter colds in humans, exhibit variable but sometimes significant seroprevalence [11], and it has been shown that pre-existing immunity can reduce the immunogenicity of replication-defective type 5 adenovirus vectors (HAdV5) used in gene delivery-based vaccine approaches. To prevent this effect, several strategies have been employed: the use of rare human adenovirus serotypes such as HAdV26, serotype switching between the first and the second vaccine doses (HAdV5 & HAdV26), or the use of chimpanzee adenovirus vectors (ChAdOx1) [12,13,14].

In contrast to HAdV5-vectored vaccines, the impact of pre-existing immunity on responses to adenovirus-derived VLPs has not been evaluated. To address this question, we genetically inserted the SARS-CoV-2 S14P5 linear neutralizing epitope [15] in the 60-mer adenovirus-VLP (Ad-VLP-S14P5). In this study, the S14P5 epitope was used as a ‘model epitope’ to assess the potential impact of the existence of adenovirus pre-existing immunity in a population. First, we tested serums from a cohort of COVID-19-infected patients and showed that the S14P5 epitope was accessible at the surface of Ad-VLP-S14P5, even in the presence of anti-vector immunity. Second, an in vivo experiment was conducted in mice to study the impact of anti-vector immunity on antibody responses to this ‘model epitope’, both with or without the addition of an adjuvant to the immunizations.

## 2. Materials and Methods

### 2.1. Baculovirus Production

MultiBac baculovirus expression system was used to produce Ad-VLP-S14P5. Sequences corresponding to S14P5 were codon optimized for insect cell production, and synthetic DNA was cloned between EcoRI and RsrII of the pACEBac1 plasmid encoding the empty Ad-VLP (Genscript, Piscataway, NJ, USA). Recombinant baculovirus was made by transfecting SF21 cells with the bacmid obtained after transposition of pACEBac1-Ad-VLP-S14P5 in EMBacY *E. coli* strain as previously described [16]. Production was performed by infecting Sf21 cells for 48 to 72 h at 27 °C in SFM medium (ThermoFisher Scientific, Waltham, MA, USA) by monitoring YFP expression. Pellets were recovered by centrifugation at 600× *g* for 5 min.

### 2.2. Ad-VLP and Ad-VLP-S14P5 Purification

Ad-VLP purification was already described [17], and same protocol was used for Ad-VLP-S14P5. Briefly, three cycles of freeze-thaw in the presence of ‘Complete’ protease inhibitor cocktail (Roche, Basel, Switzerland) of insect cell pellets were performed, and lysates were clarified by centrifugation. Lysate was loaded onto a 20–40% sucrose density gradient and ultra-centrifuged for 18 h at 4 °C on a SW41 at 41,000 RPM. The dense fractions collected at the bottom of the tubes were dialyzed against Hepes 10 mM pH 7.4, NaCl 150 mM, and then loaded onto a Macroprep Q cartridge (Bio-Rad, Hercules). After elution by a 150 to 600 mM linear NaCl gradient in Hepes 10 mM pH 7.4, Ad-VLP-S14P5-containing fractions were detected by SDS-PAGE and concentrated on Amicon (MWCO: 100 kDa) with buffer exchange to Hepes 10 mM pH 7.4, NaCl 150 mM or PBS.

### 2.3. Negative-Stain Electron Microscopy

Samples were absorbed to the clean side of a carbon film on mica, stained by uranyl acetate, and transferred to a 400-mesh copper grid. The images were taken under low dose conditions (<10 e−/Å^2^) with defocus values between 1.2 and 2.5 μm on a Tecnai 12 LaB6 electron microscope at 120 kV accelerating voltage using CD Camera Orius h1000 (Gatan, Pleasanton, California).

### 2.4. Cohort of Patients Infected by SARS-CoV-2

The cohort was composed of convalescent COVID-19 patients infected by SARS-CoV-2 in 2020, composed of 50% mild (oxygen below 2 L/min) and 50% severe (intensive care unit) cases, 6 months after hospitalization

### 2.5. Vaccination Protocol and Schedule

Vaccination experiments have been performed according to ethical guidelines. 5-week-old female Balb/c mice were purchased from Janvier (Le Genestet St. Isle, France). All the mice were immunized with Ad-VLP-S14P5 vaccine at 2-week interval (Day 0 and Day 14). Each mouse received subcutaneously the same dose of vaccine (5 µg of Ad-VLP-S14P5) in 100 µL final volume in either PBS (not adjuvanted) or adjuvanted by one volume of ADDaVax (Invivogen, Toulouse, France) in the right flank. For pre-immunized groups (II and IV), mice were immunized with 5 µg of empty Ad-VLP at D-14 (5 µg in 50 µL PBS + 50 µL ADDaVax) in the right flank. The days before each Ad-VLP-S14P5 immunization and 2 weeks after the last immunization, blood samples were collected from anesthetized mice (4% isoflurane) for serologic tests.

### 2.6. ELISA on S14P5 Peptide or Recombinant SARS-CoV-2 Spike

The S14P5 peptide was chemically synthesized and provided by Tebu-Bio. The biotin moiety was added at the C-terminus using the free NH2 group. For mice serum evaluation, plates were first coated with streptavidin (2 µg/mL, 50 µL/well) at 4 °C overnight. After washes, biotinylated S14P5 peptide (2 µg/mL) was applied for 4 h (50 µL per well), washed, and blocked with PBS-3% BSA for 1 h at room temperature. For the recombinant spike, the empty Ad-VLP and Ad-VLP-S14P5 direct coating was realized (2 µg/mL, 50 µL/well) at 4 °C overnight. After washes, wells were blocked with PBS-3% BSA for 1 h at room temperature.

Mice serums or cohort patient serums were serially diluted in PBS from 1/50 by 5-fold dilutions and incubated on plates for 1 h at room temperature. Peroxidase AffiniPure Goat Anti-Mouse IgG (heavy and light chains) from Jackson Immuno Research was used for detection (0.32 µg/mL, 50 µL by well) and incubated 1 h at room temperature. After washes with PBS Tween 0.05%, reading was performed by adding 50 µL per well of TMB (tetramethylbenzine, Merck, Darmstadt, Germany). Reaction was stopped after 1 min of incubation with addition of H_2_SO_4_ 0.5 M, and OD was measured in a microplate reader (Thermo Fisher Scientific) at 450 nm and 550 nm. Human serums were diluted similarly and then revealed using anti-Human IgG (heavy and light chains, JIR), and the area under the curve was calculated using Graph Pad.

### 2.7. Statistical Analysis

Statistics: Statistical analyses were performed using GraphPad Prism 10, employing a one-tailed Student’s *t*-test and one-way ANOVA followed by Tukey’s multiple comparisons test. A *p*-value of <0.05 was considered statistically significant. All tests were conducted alongside the respective normality and homoscedasticity tests when applicable.

## 3. Results

### 3.1. Ad-VLP and Ad-VLP-S14P5 Recognition by Serums from COVID-19-Infected Patients

**Figure 1:**
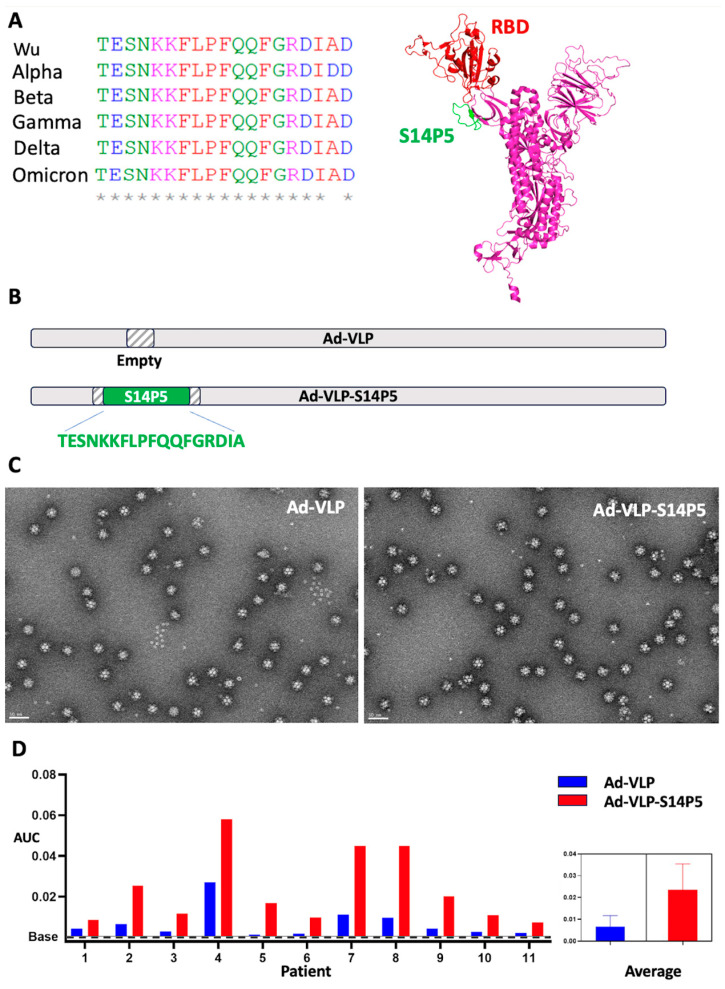
**S14P5 insertion in Ad-VLP.** (**A**) The S14P5 epitope is conserved amongst different SARS-CoV-2 variants. S14P5 (in green) is located just beneath the RBD domain (in red). * conserved residues. (**B**) The epitope is inserted in an exposed loop of the Ad-VLP, giving rise to Ad-VLP-S14P5. (**C**) Negative staining electron microscopy of the purified empty Ad-VLP and Ad-VLP-S14P5 (Bar 50 nm). (**D**) Screening by ELISA of a cohort COVID-19 infected patients serums on Ad-VLP (blue) and Ad-VLP-S14P5 (Red). Results are represented by the area under the curve (AUC).

The S14P5 linear epitope is highly conserved in the different SARS-CoV-2 variants, from the Wuhan strain to currently circulating ‘Omicron’ variants. Only one point mutation has been observed in the ‘Alpha’ variant. This epitope is located in the spike protein just above the critical ‘Receptor Binding Domain’ (RBD) involved in the ACE2 receptor recognition (Figure 1A). The sequence coding for this 19-residue peptide was genetically inserted into an exposed loop of the Ad-VLP (Figure 1B). The particle assembly was not impaired by the insertion of the epitope into the exposed loop, as shown by negative staining microscopy of the 60-mer wild-type Ad-VLP particle and the 60-mer Ad-VLP-S14P5 particle displaying the SARS-CoV-2 epitope (Figure 1C).

To verify whether the S14P5 epitope was accessible to specific antibodies, we tested serums from individuals who previously had COVID-19 for reactivity against Ad-VLP-S14P5 in comparison to wild-type Ad-VLP by ELISA. The anti-adenovirus humoral immunity was variable between donors, with some being reactive like #4 and others appearing not reactive, like #5 and #6 (Figure 1D, blue bars), reflecting the various anti-adenovirus status that can be observed in the population.

Reactivity against Ad-VLP-S14P5 was detectable even in the absence of reactivity against the wild-type VLP (#5 and #6), strongly suggesting the presence of specific anti-S14P5 antibodies. In all patients with anti-wild type VLP reactivity, the activity against Ad-VLP-S14P5 was higher, thus also reflecting the presence of specific anti-S14P5 antibodies (Figure 1D, red bars).

Overall, the data suggested that all donors in the cohort exhibited anti-S14P5 antibodies and that the linear epitope was accessible at the surface of the VLP and could, therefore, be immunogenic in this context.

### 3.2. Immunization Regimen and Assessment of Adenovirus Pre-Immunity Before Ad-VLP-S14P5 Immunizations in Mice

**Figure 2:**
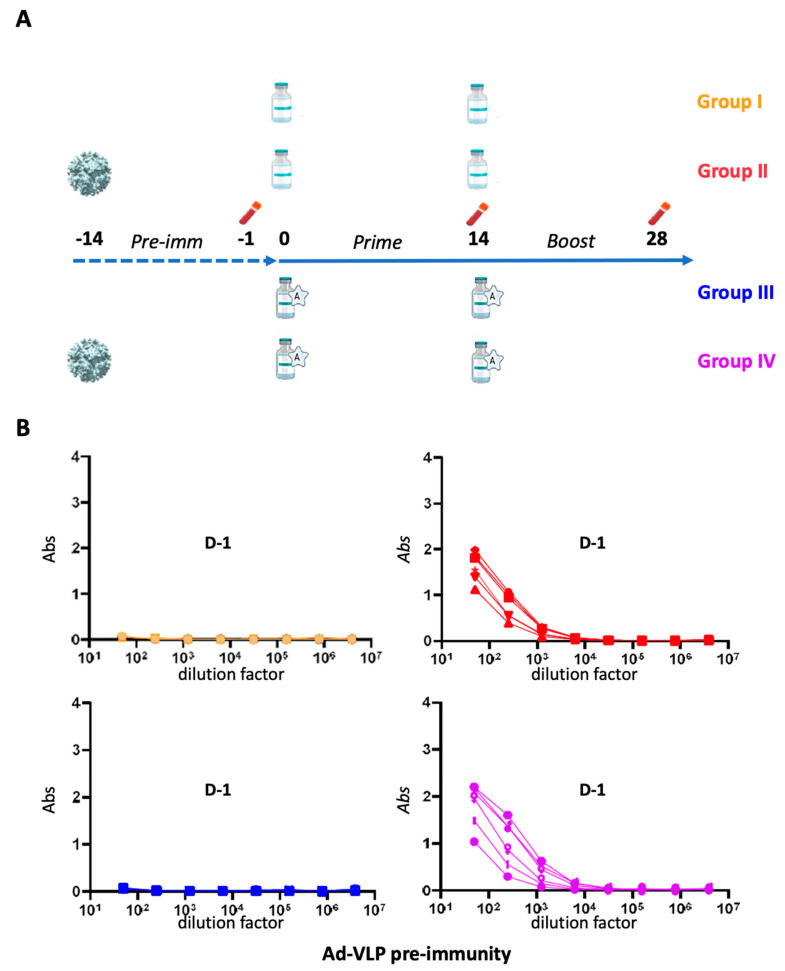
**Preimmunization of mice with empty Ad-VLP and immunization schedule**. (**A**) Four groups of mice (*n* = 6) received the same dose of Ad-VLP-S14P5 vaccine (blue vials) at D0 and D14 without (groups I and II) or with adjuvant (groups III and IV, vials labeled with ‘A-star’). Mice from groups II and IV were preimmunized with empty Ad-VLP at D-14. Blood was collected before the first (D-1), at the second (D14) injection, and at the end of the experiment (D28). (**B**) Pre-existing immunity against the vector was checked by direct ELISA against empty Ad-VLP at D-1 for all groups.

To study the role of pre-existing immunity to adenoviruses on the response to the S14P5 epitope displayed on the VLP, two groups of mice (groups II and IV) received an injection of the empty vector (*i.e.*, not displaying S14P5 epitope) two weeks before the first injection of Ad-VLP-S14P5 whereas groups I and III were kept naive (Figure 2A). To also investigate whether an adjuvant could have an effect on the response against the displayed epitope, AddaVax, an MF59-like oil-in-water adjuvant that we had previously used in another study, was injected with Ad-VLP-S14P5 in groups III and IV, while groups I and II were immunized by the same dose of Ad-VLP-S14P5 without adjuvant (Figure 2A). All the mice that had received an injection of the empty Ad-VLP (groups II and IV) had developed an immune response to the vector at D-1, as shown by ELISA against empty Ad-VLP, but was absent in groups I and III as expected (Figure 2B).

### 3.3. Association Between Pre-Existing Adenovirus Immunity and Anti-S14P5 Response in Absence of Adjuvant

**Figure 3:**
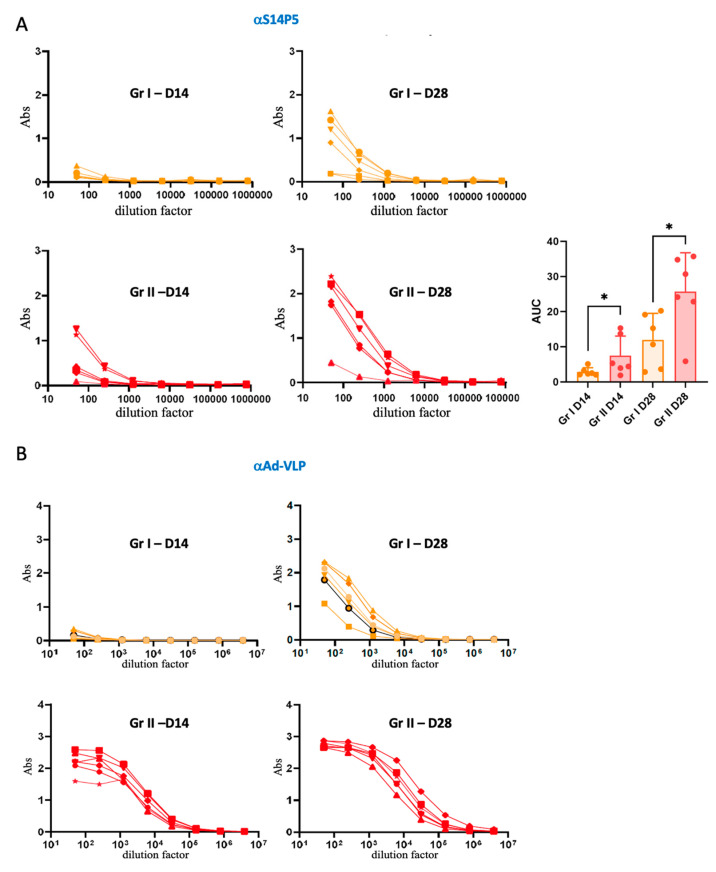
**Evaluation of anti-S14P5 and anti-vector response of mice immunized with Ad-VLP-S14P5 without adjuvant.** (**A**) ELISA was performed on biotinylated S14P5 peptide with serial dilutions of serums from groups I and II collected at D14 and D28. Statistical analysis was performed by calculating the area under the curve (AUC) of mice from each group using unpaired T test (one-tailed) D14 * *p* = 0.0406, and D28 * *p* = 0.0156. (**B**) Similar experiment on empty Ad-VLP to determine the corresponding anti-vector response.

In the case of recombinant adenoviruses used as vaccine vectors, immunity to the vector is often a problem, necessitating the use of vectors from low-prevalence serotypes. To assess the impact of anti-vector pre-existing immunity on vaccination with adenovirus-derived VLPs in the absence of adjuvant, naive (group I) or adenovirus-primed mice (group II) were immunized with Ad-VLP-S14P5 alone. Serums collected two weeks after the first and second immunization (D14 and D28) were analyzed by ELISA using a biotinylated S14-P5 synthetic peptide. At day 14, while the anti-S14P5 response was nearly undetectable in adenovirus-naive mice, its onset was already observable in mice with anti-vector immunity (Figure 3A, red curves). After the boost injection (D28), an anti-S14P5 response was observed in mice from both groups, but the response was significantly better in group II (*i.e.*, pre-immunized with empty Ad-VLP).

When looking at the anti-vector response on day 14, it was still nearly non-existent in group I (Figure 3B, orange lines), whereas it was strong in all animals in group II (Figure 3B, red lines). At day 28, Abs to Ad-VLP could finally be detected in group I, while titers increased in group II. Overall, in group I, responses against Ad-VLP paralleled those against S14P5.

Overall, anti-vector immunity was, therefore, not detrimental to the immune response against the epitope displayed by the VLP, but on the contrary, significantly increased its recognition and processing by the immune system.

### 3.4. Association Between Pre-Existing Adenovirus Immunity and the Anti-S14P5 Response in the Presence of Adjuvant

**Figure 4:**
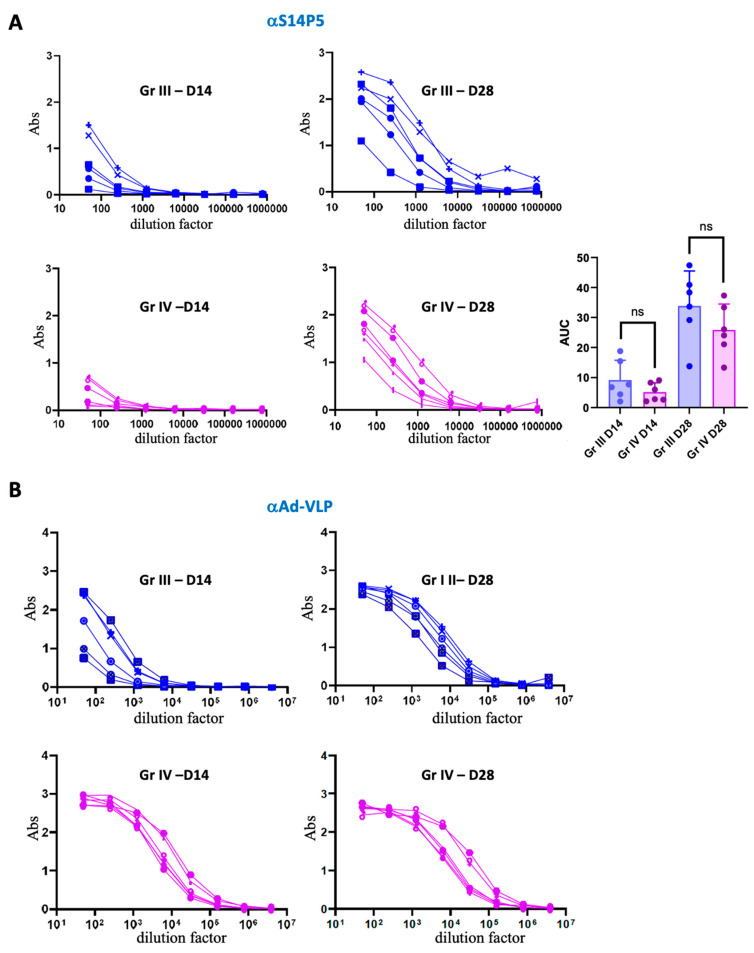
**Evaluation of S14P5 and anti-vector response of mice immunized with adjuvanted Ad-VLP-S14P5.** (**A**) ELISA was performed on biotinylated S14P5 peptide with serial dilution of serums from groups III and IV collected at D14 and D28. Statistical analysis was performed by calculating the area under the curve (AUC) of mice from each group using unpaired *t* test (one-tailed); ns: not significant. (**B**) Similar experiment on empty Ad-VLP to determine the corresponding anti-vector response.

We then evaluated the impact of anti-vector pre-existing immunity on vaccination with adenovirus-derived VLPs in the presence of adjuvant by comparing responses to Ad-VLP-S14P5 administered with AddaVax in naive (group III) and adenovirus-primed mice (group IV). At day 14, the anti-S14P5 response (Figure 4A) was detectable in both groups. This contrasted with results obtained in the absence of adjuvant, where pre-immunization was necessary to obtain a response against S14P5 at this early time point (Figure 3A).

On day 28, both groups exhibited a strong response against S14P5. In contrast to immunization in the absence of adjuvant (Figure 3A), robust responses were observed even in the absence of pre-immunization (Figure 4A). Notably, there was a trend toward a stronger response in non-pre-immunized mice compared to pre-immunized ones, although the difference was not statistically significant (Figure 4A).

Regarding anti-vector immunity, at day 14, as expected, responses were stronger in the pre-immunized group. Of note, the addition of an adjuvant to the Ad-VLP-S14P5 particle markedly increased the early anti-Ad-VLP response (Figure 4B, D14), whereas it was nearly absent at day 14 without adjuvant (Figure 3B, left top panel). At day 28, anti-Ad-VLP antibody titers were high in both groups (Figure 4B, D28) and notably higher than in animals without pre-existing immunity, immunized without adjuvant (Figure 3B, Gr I, D28).

Overall, the addition of AddaVax results in the best absolute response against the epitope of interest in mice with no pre-existing immunity (Figure 4A, Gr III), as compared to the three other groups. An antagonist effect is observed in the absence or presence of adjuvant since the pre-existing immunity against the vector, which was beneficial in the previous experiment (*i.e.*, without AddaVax), is replaced by a trend to a lower response against the epitope of interest upon adjuvant addition.

### 3.5. Functionality of Anti-S14P5 Recognition on the Recombinant Trimeric SARS-CoV-2 Spike

**Figure 5:**
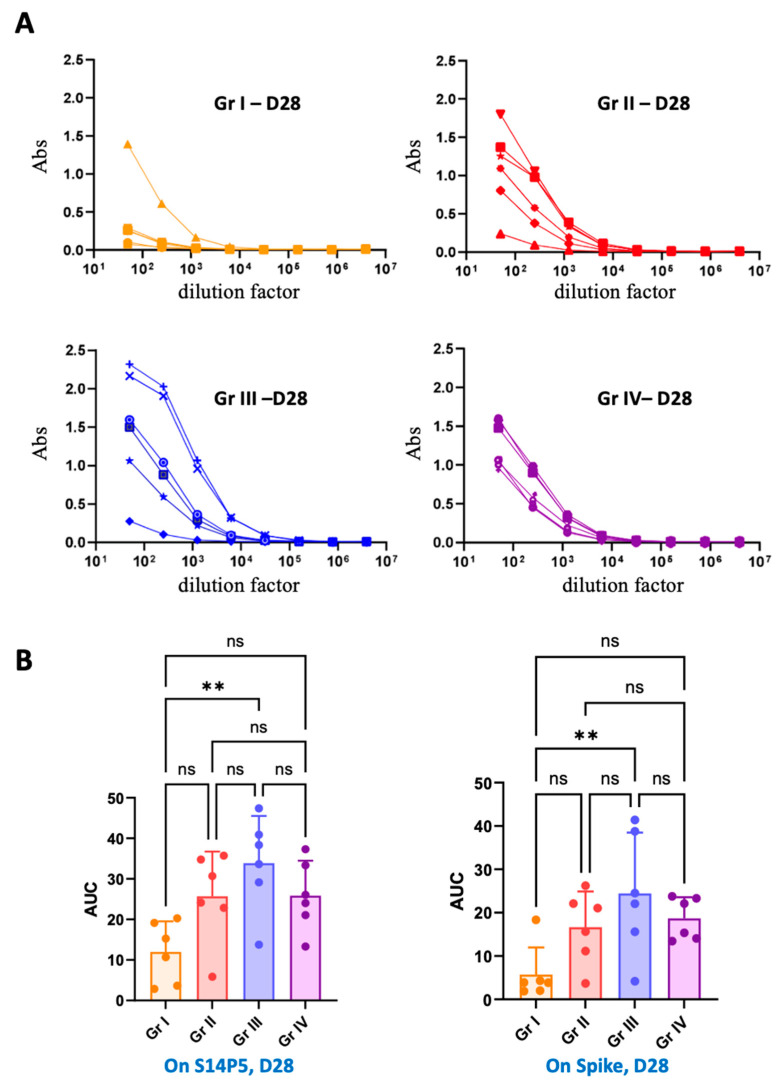
**Recognition of recombinant trimeric spike by the four groups.** (**A**) ELISA was performed on soluble trimeric recombinant spike with serial dilution of serums from the four groups collected at D28. (**B**) Correlation between the result obtained on the biotinylated S14P5 and the recombinant spike. Statistical analysis was performed by calculating the area under the curve (AUC) of mice from each group on S14P5 at D28 (left panel) and on recombinant spike at D28 (right panel) using one-way ANOVA followed by Tukey’s multiple comparisons test ** *p* < 0.01; ns: not significant.

In order to determine whether the antibodies produced in mice upon Ad-VLP-S14P5 immunizations could recognize the epitope S14P5 within the context of the recombinant trimeric spike protein, day 28 animal serums were tested by ELISA against this protein. Serums from each group recognized the recombinant spike protein, showing that antibodies induced by Ad-VLP-S14P5 were active against the biological target (Figure 5A). Interestingly, a strong correlation was observed between the ELISA performed on biotinylated S14P5 (Figure 3A and Figure 4A) and on the recombinant trimeric spike, with group I giving the lowest titers and group III giving the highest (Figure 5B).

## 4. Discussion

A major problem for the development of pan-sarbecovirus vaccines is the rapidity with which viruses adapt to immune systems, creating escape variants [18]. To alleviate this problem, the existence of the virus’s Achilles heel is sought in order to find immunogenic zones that the virus cannot easily mutate. The S14P5 epitope proved to be an interesting target in 2020, producing neutralizing antibodies against the Wuhan strain [15]. Interestingly, four years later, S14P5 is still conserved in currently circulating mutants such as the Omicron variants (Figure 1A). Thus, we decided to use S14P5 as a ‘model epitope’ in our study. S14P5 sequence was genetically inserted (Figure 1B) into an adenovirus-derived 60-mer VLP previously reported to trigger a humoral response against a linear Chikungunya epitope [8]. Insertion of this epitope in an exposed loop did not affect the scaffold, as seen by electron microscopy (Figure 1C). In contrast with our previous study with E2P3, for which a specific cleavage at the N-ter of the epitope (thanks to the insertion of a TEV site) was required before immunization to induce an efficient humoral response, S14P5 proved to be efficient in the internal loop of the VLP without prior proteolytic treatment. This could be explained by the fact that the E2P3 epitope is exposed after furin cleavage of the Chikungunya virus E2 protein, whereas S14P5 does not require the SARS-CoV2 spike furin cleavage to be exposed.

Knowing that adenovirus is a common pathogen that most people contract during their lives [19], the role played by this immunity needs to be taken into account. In our study, a cohort of eleven COVID-19-recovering patients was tested for the presence of pre-existing immunity against the vector. The results show great differences in adenovirus preexisting immunity amongst patients, highlighting the importance of studying its influence on the epitope displayed by Ad-VLP (Figure 1D). Moreover, the data also confirmed the detection of specific anti-S14P5 antibodies in all the COVID-19 patients, reflecting its universal recognition during SARS-CoV-2 infection. More importantly for our study, the detection of S14P5 inserted in an internal loop of the VLP demonstrates that the linear epitope was accessible at the surface of the VLP even decorated by human anti-vector antibodies and could, therefore, be immunogenic whatever the adenovirus pre-existing immunity context.

It is well known that the role played by pre-existing immunity to a vector is crucial when using recombinant viral vectors, as spectacularly shown during the COVID-19 crisis. Indeed, the first vaccines developed were derived from recombinant adenoviruses and had to be derived from the rare human (HAdV-26) or from animal-derived adenovirus serotypes (Chimpanzee Adenovirus: ChAdOx1) [12,13,14]. When using recombinant viruses which serve as trojan horses, the gene encoding the antigen of interest must be absolutely delivered into the cell, thus requiring viral internalization [20]. When using VLPs, the antigen being displayed by the particle for direct presentation to the immune system, the outcome could be different, but less is known about the role played by vector pre-existing immunity.

To provide information, an experiment was carried out in mice either naive to the adenoviral-derived VLP or previously pre-immunized with the empty Ad-VLP (Figure 2A). As expected, anti-vector antibodies were not found at D-1 in mice from the naive groups, whereas all the Ad-VLP adjuvanted pre-immunized mice were positive after a single immunization. This status being checked, we decided to study Ad-VLP-S14P5 immunization in the presence or absence of an adjuvant.

First, in the absence of any adjuvant, the humoral response against the epitope of interest was studied two weeks after the first immunization and two weeks after the boost. Unexpectedly, the anti-S14P5 immune response was detectable in mice with anti-vector immunity as early as the first injection, whereas no significant response was observed in adenovirus-naive mice (Figure 3A). This trend was confirmed after the second injection, albeit less markedly. One hypothesis could be that the Fc fragment of the anti-adenovirus antibodies bound to the Ad-VLP-S14P5 particle allows better uptake of the immune complex in the APCs via the γFc receptors [21,22], triggering a faster and more efficient response against the displayed epitope. This result demonstrates that minimizing pre-existing immunity against VLP would not always be desirable [23].

If VLPs can be used in the absence of adjuvant due to their repetitive motifs known to cluster B-Cell Receptors (BCR) [24], they are nevertheless often supplemented with adjuvant [25]. To investigate whether our previous observations were also observed in the presence of an adjuvant, a similar experiment was carried out in the presence of ADDaVax, an oil-in-water MF-59-like emulsion [26,27], which we had used successfully in a previous SARS-CoV-2 study [28]. Overall, the addition of this adjuvant enabled a response from the first injection with a boost at the second injection, whatever the adenovirus immunological status of the mice. Contrary to what was observed in the absence of adjuvant, pre-existing immunity against the vector was no longer beneficial and a detrimental trend was even observed upon adjuvant addition (Figure 4A).

We might wonder whether the vaccine dose used could have an effect on the immunization outcome. In our study, 5 micrograms of Ad-VLP-S14P5 were used per immunization, which is in the upper range for this type of study. It should be noted that in another study using a nanoparticle displaying the receptor binding domain of SARS-CoV2, the authors showed in BALB/c mice immunized with AddaVax that doses of 1 microgram or 0.1 micrograms of immunogen had a minor effect on the immune response [29]. This suggests that the effect observed in our study is probably unrelated to the dose of immunogen used.

S14P5 is a 19-residue epitope that was inserted into an exposed flexible loop of the Ad-VLP. To investigate whether the antibodies produced in mice were functional in a context closer to reality, an ELISA was carried out with recombinant trimeric spike protein. The data showed that serums from vaccinated mice were capable of recognizing the viral glycoprotein and, thus, that S14P5 is accessible to antibodies despite its location beneath the RBD domain (Figure 1A and Figure 5A). Of note, a good correlation is seen with the results obtained on the synthetic S14P5 peptide, confirming the superiority of group III compared to the three other ones (Figure 5B).

Overall, even if our study is limited to the formulation with AddaVax and would benefit from being extended to other adjuvants, the data clearly show that particular care needs to be taken when designing a vaccine regimen using VLPs. Based on our results, it would have been interesting to carry out an initial immunization in the presence of an adjuvant to elicit a response against both the epitope of interest and the vaccine platform. The second immunization could then be carried out in the absence of an adjuvant to see if the anti-vector antibodies would play a potentiating effect on the epitope of interest. If we transpose our results to humans, a segmentation of recipients could be of interest. We could imagine that people with no anti-adenovirus immunity would benefit from being immunized in the presence of an adjuvant, while those with anti-vector antibodies could receive the non-adjuvanted vaccine. Even if this transposition is purely hypothetical, our study confirms that vaccination protocol design is of high importance in the outcome of immunization when using VLPs. Our study is a step forward in both the understanding and better usage of VLP vaccines.

## Data Availability

Data can be obtained upon request.
